# Conception and pilot testing of a self-management health application for patients with pollen-related allergic rhinitis and allergic asthma-the APOLLO app

**DOI:** 10.1038/s41598-023-48540-4

**Published:** 2023-12-07

**Authors:** V. Landesberger, K. Grenzebach, F. Schreiber, D. Nowak, M. Gröger, E. Oppel, B. Schaub, L. E. French, S. Kutzora, C. Quartucci, C. Herr, S. Heinze

**Affiliations:** 1 Bavarian Health and Food Safety Authority, Munich/Oberschleißheim/Erlangen, Germany; 2 Institute and Clinic for Occupational, Social and Environmental Medicine, University Hospital, LMU Munich, Munich, Germany; 3 Comprehensive Pneumology Center (CPC) Munich, Member of the German Center of Lung Research (DZL), Munich, Germany; 4 Department of Otorhinolaryngology, University Hospital, LMU Munich, Munich, Germany; 5 Department of Dermatology and Allergy, University Hospital, LMU Munich, Munich, Germany; 6 LMU Munich, University Children’s Hospital, Munich, Germany; 7 Member of the German Center of Lung Research (DZL), Munich, Germany

**Keywords:** Epidemiology, Health services, Public health

## Abstract

It has been shown that pollen information services are an important self-management tool for patients with pollen-related allergic rhinitis (AR) and allergic asthma (AA). This study aimed to design an online application for patients with AR and AA, which supports patients to better manage their disease as well as to evaluate the app and present the first results of the pilot study. The pollen data were obtained from the electronic pollen information network of Bavaria, Germany. Participants were asked to fill in their allergy-related complaints in the app over a 60-day period. Subsequently, the app was evaluated. Indices and diagrams visualized the participants’ individual complaints as well as the daily pollen concentration in the air. In order to motivate participants to complete the app on a daily basis, we used elements of gamification. Two thirds of the participants (N = 46) reported feeling better informed about pollen counts and their allergy when using the app. The app's simple and comprehensible design was rated positively. More than 80% of the participants would recommend the app to their family and friends. The app can be a tool for patients with AR and AA to better understand their disease.

## Introduction

Allergic diseases are a major global public health issue and their prevalence continues to increase dramatically worldwide, even if the prevalence is stabilizing in some western countries^1–3^. An allergy is an exuberant reaction of the immune system to typically harmless substances. These allergy-triggering substances are called allergens^4^. Pollen are one of the most common allergens.

Pollen-associated allergic diseases include respiratory diseases such as allergic rhinitis (AR, synonyms: hay fever, pollinosis) and allergic asthma (AA). According to the World Allergy Organization (WAO), AR affects 10–30% of the population worldwide^5^. A study on the health of adults in Germany found lifetime prevalence for bronchial asthma to be 8.6%, and for hay fever 14.8%^6^. Due to climate change, these numbers are likely to increase in the future, since, rising temperatures and carbon dioxide levels affect phenology and distribution of pollen species among other things, leading to a longer pollen season and increased pollen allergenicity and quantity^7–9^.

The symptoms of AR and AA affect allergy patients in many different ways^10^. Physical symptoms in allergic patients include, among others, nasal congestion, sneezing, itchy-watery and swollen eyes^11^. Additionally, AR can have an influence on quality of life, sleep, usual performance of daily activities and work productivity^12^. There are pharmacological as well as non-pharmacological measures to prevent and control allergic symptoms in pollen allergy. Many patients rely on pharmacotherapy, such as oral or topical antihistamines and intranasal corticosteroids to manage their pollen allergy^13^. Although drug therapy usually has a good and rapid effect, it may be associated with various side effects such as impaired ability to focus and an increased need for sleep^14^. Immunotherapy on the other hand can change the course of allergic rhinitis, if patients are willing to undergo time-consuming treatment^13,15^. In general, some patients have lost trust in their health care professionals (HCPs) or prescribed medications, causing them to experiment on their own and trying out different medications^16,17^. That also leads to a more frequent use of non-pharmacological measures such as avoiding allergens and enhanced self-management of pollen allergy e.g. using pollen diaries and apps.

It has been shown that pollen forecasts and pollen information services are important tools for allergen avoidance, management and treatment of pollen allergies^18^. Information of current pollen counts, pollen forecasts and general information on pollen allergy are provided as websites, newsletters and apps.

To combine the advantages of an online application with pollen monitoring and self-management of pollen allergies, we have developed the APOLLO app. The app is designed to help patients with pollen-related allergic rhinitis and asthma to better understand and control their own allergy using the app’s individualised functions. To test the APOLLO app, the APOLLO study was initiated in 2021.

Firstly, the aim of this pilot study was to provide an overview of how the app was designed. Secondly, the aim was to evaluate the feasibility and the benefit of the app for the participants as well as to present the results of the pilot testing.

## Results

### Conception of the APOLLO app

**Figure 1:**
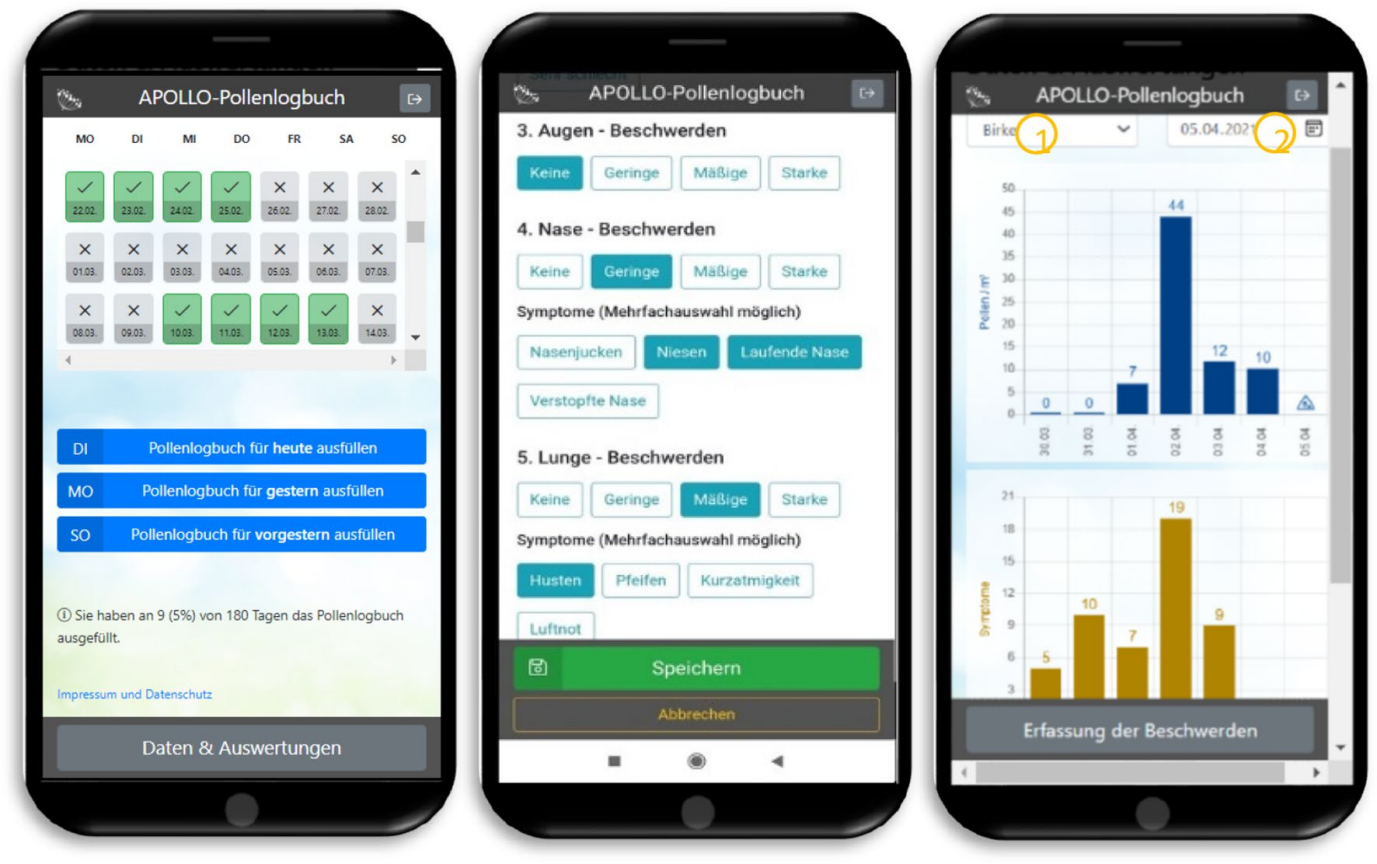
Screenshot of the calendar overview of the 60-day study period (1), the daily questionnaire to assess allergic complaints (2) and one example of the diagram of daily pollen concentration in the air (3) in the last seven days. X-axis: last seven days (Pollen concentrations of the selected location are displayed) Y-axis: pollen per m^3^. In the diagrams, participants could choose between different pollen types^1^, measurement locations and calendar days^2^.

The final version of the app consisted of three sections: (1) A calendar overview of the 60-day study period, (2) a questionnaire where patients were able to enter their complaints (physical symptoms and impairments in everyday life), and (3) diagrams on pollen concentration, physical symptoms and impairments in everyday life due to pollen allergy based on the daily questionnaire (Fig. 1).

As soon as the first version of the app was completed, we conducted a pre-test with a sample of seven test-participants consisting of employees in the public health service. Based on the feedback from the pre-testers, the app was adapted accordingly.

The results of the literature search identified the most common complaints of AR and AA patients. These complaints were entered into the app for selection (Table 1).

**Table 1:** Overview of queried complaints, respective levels of severity and indices due to the participant’s allergy.

	Eyes		Nose		Lungs				
	Severity		Severity		Severity				
Allergy-related physical symptoms							Symptom Index (SI): sum of amount of symptoms in the eyes/nose/lungs and the level of severity The SI is the sum of all indices (index eyes + index nose + index lungs)		
	None	+ 0	None	+ 0	None	+ 0			
	Slight	+ 1	Slight	+ 1	Slight	+ 1			
	Moderate	+ 2	Moderate	+ 2	Moderate	+ 2			
	Severe	+ 3	Severe	+ 3	Severe	+ 3			
	Symptom		Symptom		Symptom				
	Itchiness	+ 1	Itching	+ 1	Coughing	+ 1			
	Swollen eyes	+ 1	Sneezing	+ 1	Wheezing	+ 1			
	Redness	+ 1	Watery nose	+ 1	Shortness of breath	+ 1			
	Tearing	+ 1	Nasal congestion	+ 1	Dyspnoea	+ 1			
Index	0–7		0–7		0–7		0–21		

	Impairment of general performance		Impairment of sleep		Impairment of social contacts		Impairment of activities/hobbies		
	Severity		Severity		Severity		Severity		
Allergy-related impairments of everyday life	Not at all	+ 0	Not at all	+ 0	Not at all	+ 0	Not at all	+ 0	Index of impairment in everyday life (II): sum of the amount of impairments (1–4) and the severity of impairments (0–3)
	Somewhat	+ 1	Somewhat	+ 1	Somewhat	+ 1	Somewhat	+ 1	
	Quite	+ 2	Quite	+ 2	Quite	+ 2	Quite	+ 2	
	Extremely	+ 3	Extremely	+ 3	Extremely	+ 3	Extremely	+ 3	
Index	0–3		0–3		0–3		0–3		0–12

Multiple selection was possible. For each physical symptom and impairment in everyday life, participants could choose one of four severity levels. We formed an index of the number and severity of physical symptoms (Symptom Index, SI) as well as of the number and severity of impairment in everyday life (Impairment Index, II) (Table 1). Participants were asked to complete the app daily over a period of 60 days (one entry every day). Based on this data, the SI and II were calculated and displayed in a diagram. In addition to these two diagrams, participants were also shown the daily pollen concentration in a diagram (Fig. 1).

To identify the appropriate pollen monitor for each participant on a daily basis, participants were able to select one of the eight pollen monitors that are part of ePIN closest to their location on that day. Additionally, there was the option “not in Bavaria”, which was essential for being on holidays, business trips or similar. On these days, when “not in Bavaria” was selected, no pollen concentration could be determined. Participants had the opportunity to select the daily average pollen count of eight different pollen species (Alnus, Ambrosia, Artemisia, Betula, Cupressaceae, Fraxinus, Poaceae and Urticaceae). If the participants had indicated an allergy to another pollen species in Q1, these species were also available for selection.

Participants were asked to complete the APOLLO app subsequently for 60 days, which requires a high level of adherence. In order to motivate participants to complete the app on a daily basis, we developed a concept for gamification. Firstly, participants were offered a 20 Euros voucher if they completed the diary on at least 51 out of 60 days (85%). Secondly, the app showed a calendar overview of all past days in which the app could be filled in until the current day. Days on which the app was completed were marked with a green tick. Days on which the app was not completed at all or only incompletely were greyed out in the calendar view and marked with an x. A percentage at the bottom of the calendar showed how many of the 60 days in the app had already been completed (Fig. 1). Thus, the participants were daily informed about the likelihood of receiving the incentive. Thirdly, if the app was not completed at all or only incompletely on a particular day, participants were reminded to complete the app at 8 pm via push notification. The participants could turn off the push notifications individually.

### Results of pilot testing

**Figure 2:**
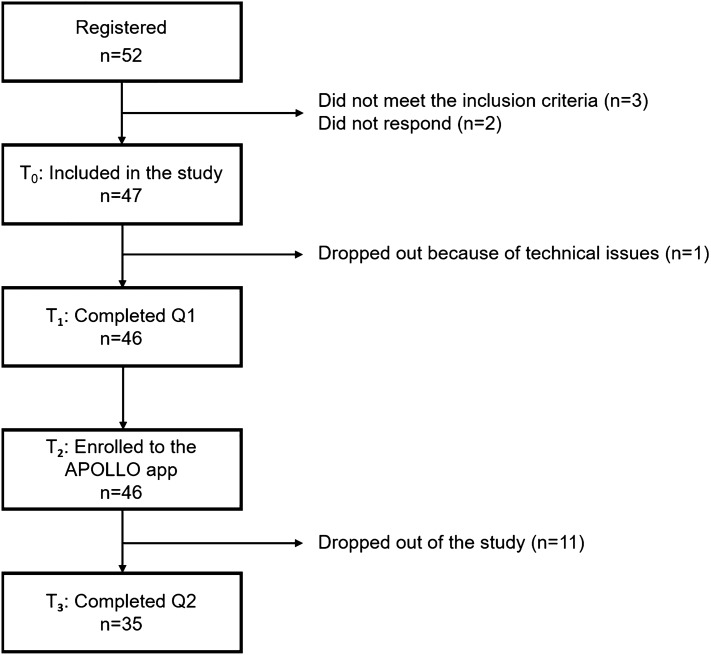
Adherence of participants throughout the APOLLO study period. T_1_: participants included in the study. T_2_: Participants enrolled to the APOLLO app. T_3_: Participants completed all questionnaires.

Overall, 52 participants registered for the pilot study. Forty-seven participants met the inclusion criteria; one person was excluded due to technical issues (Fig. 2). The remaining 46 participants completed Q1 and were enrolled for testing the APOLLO app.

Table 2 presents the characteristics of this sample. All participants were living in Bavaria, Germany. About two thirds of the participants were female. Most participants were of German origin, had a good general health and a university degree. The participants reported being allergic to the following pollen: birch (n = 36), hazel (n = 29), alder (n = 26), ash (n = 11), grasses (n = 36), rye (n = 19), mugwort (n = 11), ragweed (n = 5), plantain (n = 3), beech (n = 1) olive tree (n = 1) and timothy (n = 1). Most of the participants suffered from more than one allergy. Six participants tested the APOLLO app without knowing which specific pollen allergy they had. About one third of the participants (n = 15) had at least one doctor's visit per month in the last year due to their pollen allergy.

**Table 2:** Descriptive analysis of study population (n = 46).

Characteristics (N = 46)	Values n (%)
Recruitment of participants	
Via department at LMU Munich	12 (26.1)
Via websites and lectures	10 (21.7)
No information about recruitment	24 (52.2)
Gender	
Female	28 (60.9)
Male	18 (39.1)
Age	
Mean Age (Min–Max; SD)	34 (14–65;12.2)
Migration background	
German	43 (93.5)
Others	2 (4,4)
Unknown	1 (2.2)
Graduation	
University entrance qualification	41 (89.1)
Currently in school education	2 (4.3)
Secondary school diploma	2 (4.3)
Unknown	1 (2.1)
General health condition	
Very good	23 (50.0)
Good	22 (47.8)
Bad	1 (2.2)
Very bad	0 (0.0)
Asthma	
Yes	17 (37.0)
No	28 (60.9)
Unknown	1 (2.2)
Atopic dermatitis	
Yes	16 (34.8)
No	29 (63.0)
Unknown	1 (2.2)
Absenteeism*	
At least 1 time per year	8 (17.4)
Less than 1 time per year	38 (82.6)
Presentism*	
At least 1 time per year	19 (42.2)
Less than 1 time per year	27 (57.8)

*Absenteeism and presentism due to the participants’ pollen allergy.

**Figure 3:**
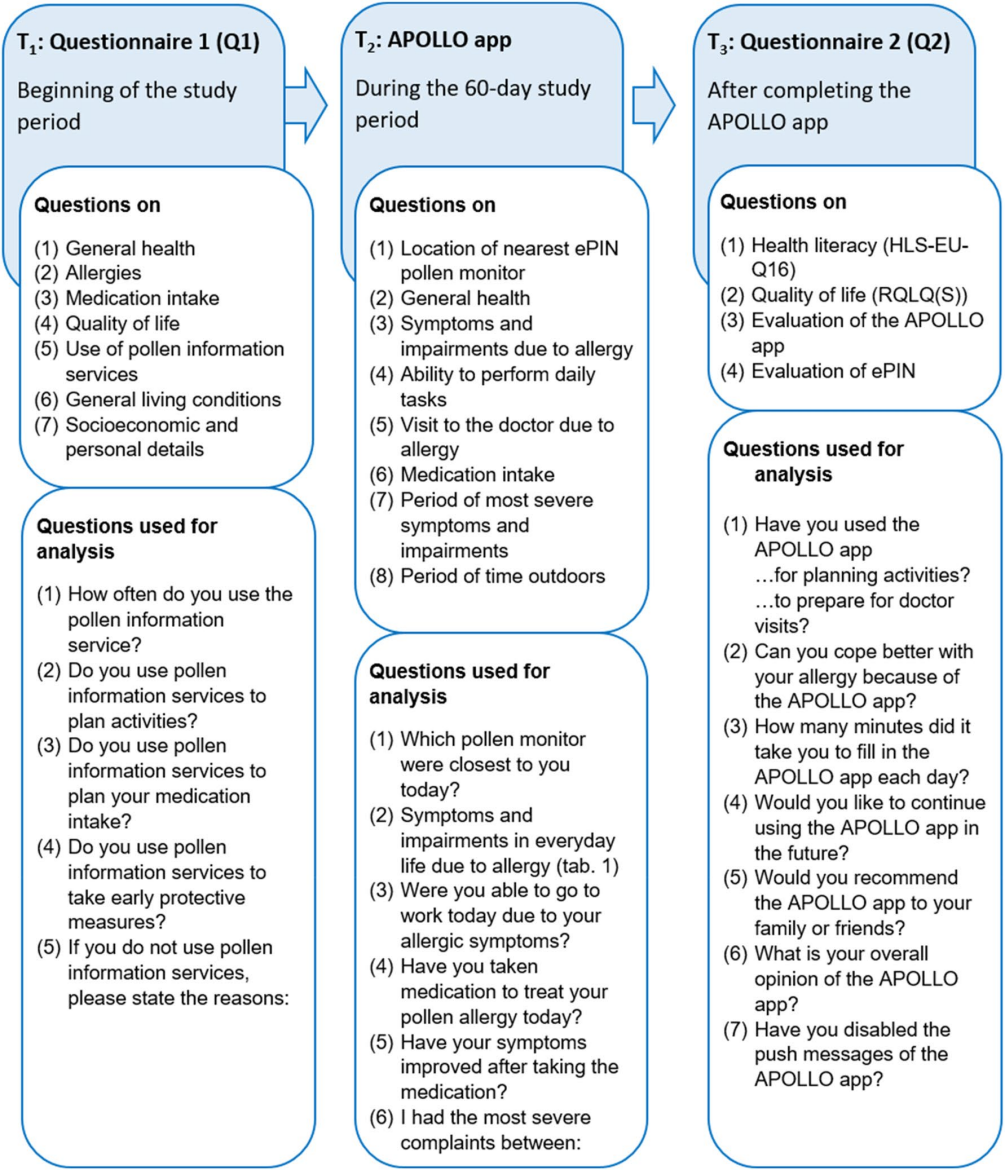
Overview of the study course and the questions used in the APOLLO study [T_1_ − T_3_].

Of the 46 participants, 76.1% (n = 35), completed all three parts of the study: Q1, the APOLLO app and Q2 (Fig. 3). In order to receive the incentive, participants had to complete the app on 85% of the days, which was the case in 69.6% (n = 32) of the participants. Neither pre-reported severity of symptoms nor the diagnosis of asthma had a significant effect obtaining the required number of completed entries (85%). The same applies to health literacy.

A total of 2,103 entries were entered into the APOLLO app by 46 participants. Data of the pollen monitor in Munich was used most frequently (72.8%, 1,530/2,103 days). In about 10% of the entries, participants had been outside Bavaria, and therefore, no pollen concentrations could be provided for these days. About one-third of the participants (32.6%) used the option to select more than one pollen monitor within the app period.

The most severe complaints (6 h intervals) of the day occurred between noon, 12 and 6 pm (on 840/ 2,103 days, 39.9%), as well as in the evening, between 6 pm and midnight (on 829/ 2,103 days, 39.3%). The most severe physical symptoms affected nose and eyes, and the most severe impairments in daily life were related to sleep patterns and performance (Table. 3).

**Table 3:** Occurrence of participants' complaints compared to all completed diary entries respectively.

			At least once* n (%)	At least in half of the entries** n (%)
Complaints	Physical symptoms	Eyes	38 (92.7)	13 (31.7)
		Nose	40 (97.6)	24 (58.5)
		Lung	31 (73.8)	9 (16.7)
	Impairments in everyday life	Performance of daily activities	36 (85.7)	7 (28.6)
		Sleeping behaviour	38 (90.5)	13 (31.0)
		Social contacts	27 (64.3)	5 (11.9)
		Hobbies	30 (73.2)	7 (17.1)

*Occurred at least once of one participants entry.

**Occurred at least at 50% of one participants entry.

On 4.0% (85/ 2,103 days) of the analysed days, it was not possible for the participants to go to work regularly. Regarding medication use, participants reported taking medication due to pollen allergy on 33.0% (694/ 2,103 days) of the days. Medication usually resulted in improvement of complaints (93.9%, 590/694 days).

Almost half of the participants (47.8%, n = 22) had never used pollen information services, 13.0% (n = 6) used these services once a month or less frequently and almost 40% (n = 18) of the participants used pollen information services at least once a week. There was no significant difference in reporting the use of pollen information services among participants diagnosed with asthma compared to participants without asthma. One third of these participants (36.4%, n = 8) used pollen information services to plan their activities, almost half of those (45.5%, n = 10) to plan their medication and 45.5% (n = 10) to use early protective measures. More severe pre-reported physical symptoms correlated with more frequent use of pollen information services (r = 0.33; p = 0.025), but more pre-reported physical symptoms did not correlate with the assessed quality of pollen information services. Half of the participants who had never used pollen information services, reported the reason to be, that they did not know about its existence (n = 11).

### Results of evaluation

The results of the evaluation of the diagrams can be seen in Table 4. In addition, two thirds of the participants felt better informed about the pollen count and their own allergy with the app (61.8%, n = 21). One third of the participants felt that the app made them feel better prepared for a consultation with their doctor (32.3%, n = 11) and another third of the participants felt that the app made them cope better with their allergy (38.2%, n = 13). However, the app increased the quality of life of only 8.8% (n = 3) of the participants.

**Table 4:**
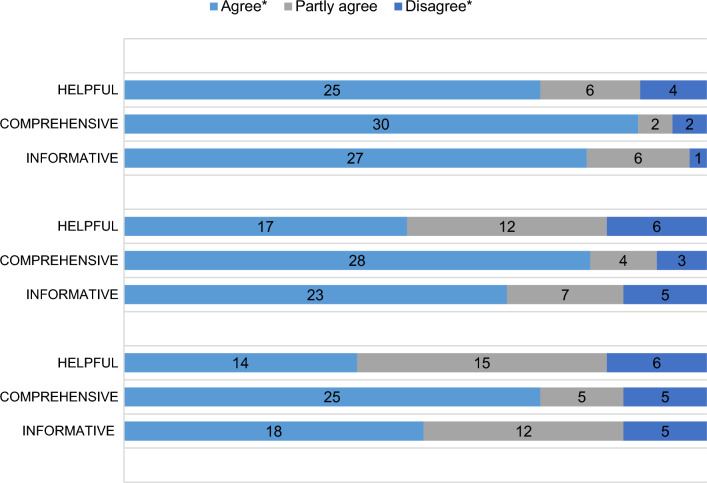
Evaluation of the diagrams in the APOLLO app.

*The categories “agree” and “tend to agree” as well as the categories “disagree” and “tend to disagree” have been grouped.

The majority of the participants indicated that they mostly had assessed their physical symptoms correctly (69.7%, n = 23), as well as their impairments in everyday life (75.8%, n = 25) in a retrospective comparison. Push notifications were used by all the participants, these notifications helped most of the participants to fill in the APOLLO app on a daily basis (97.14%, n = 34), only two participants (5.7%) used the push messages to take their medication. The APOLLO app was rated overall as good by 97.2% (n = 34) of the participants, 82.9% (n = 29) of the participants would recommend the APOLLO app to family and friends and 54.3% (n = 19) of the participants would at least also use the APOLLO app in the future. Asthmatics did not rate the APOLLO app differently compared to non-asthmatics.

The severity of pre-reported symptoms has a small but significant positive effect on the assessed quality of the APOLLO app, F (1; 32) = 5.96, *p* = 0.023. On average, 2 min per day were needed to fill out the APOLLO app (mean = 2 min; Range: 1–10).

The qualitative analysis showed the following results: the comprehensive and simple layout of the app was positively highlighted (n = 11). The participants expressed positive feedback on the diagrams provided on pollen concentrations in the air, physical symptoms as well as on impairments in everyday life (n = 8). Three of the participants mentioned that they dealt more with their allergy because of using the APOLLO app, which had a positive effect on their health. In addition, two participants used the app to determine which pollen species caused the majority of their complaints. In the further development of the app, the participants would wish for a pollen forecast for the next days (n = 3), as well as more than eight pollen monitors in the state of Bavaria (n = 2).

## Discussion

### General findings

The APOLLO app supports patients with pollen-related allergic rhinitis and allergic asthma to track their daily allergy-related complaints and medications, to visualize their symptoms and impairments in everyday life, and to monitor pollen concentrations in the air. For this purpose, the app provides daily pollen data from eight pollen-monitoring sites in Bavaria.

The majority of the participants completed the APOLLO app over a period of 60 days, which might be due to the gamification (incentive, push notification, calendar, percentage of filled in entries). Almost all participants reported being reminded to complete the APOLLO app with help of daily push messages. An even higher adherence might have been achieved if HCPs prescribed the app to their patients^19,20^. In other studies, significantly lower participation rates were achieved without a reminder function (push message, SMS)^21–24^. The results show that user motivation is an important factor to consider when designing a health app.

The pilot testing of the APOLLO app demonstrated that participants most frequently suffered from nasal and eye-related symptoms, impaired sleeping behaviour and impaired performance of daily activities. These results are also in line with the results of other studies^24–27^. To our knowledge, the APOLLO app is the first app to ask its participants about their impairments in everyday life on a daily basis. It shows that in addition to physical symptoms, impairments in the daily life of AR and AA patients need to be retrieved and treated to the same extent. The indices, which were formed from the allergy-related physical symptoms and impairments in everyday life, showed the users of the app the severity of their complaints on a daily basis compared to previous days. This function is also not available in already existing allergy apps. The participants generally rated the APOLLO app positively. In particular, the visualization of the diagrams on the pollen concentration in the air, as well as on the reported physical symptoms and impairments in everyday life were rated as helpful and informative. Many patients feel that they do not receive sufficient advice from their HCP and control their medication intake themselves^17,28,29^. By visualizing the complaints and displaying the pollen concentrations, patients can learn to self-identify their needs and determine individual threshold values for themselves. If a high concentration of pollen in the air is displayed, the allergy patient can spend more time indoors or adjust their medication intake. Other studies have already shown that patients also adjust their medication to the severity of their symptoms^30^. Our results provide indications that half of the participants felt better informed about their allergy and the pollen count with the APOLLO app. However, the pilot study was not able to show that the APOLLO app improved the participants' quality of life. Yet another study^23^ was able to show an improvement in quality of life through the use of their app. Further analyses with a larger sample size are needed to verify these results.

Nevertheless, allergy apps have not been evaluated frequently^31^. Participants who pre-reported severe allergy related symptoms rated the app better than participants with less pre-reported physical symptoms did. Similar results were also reported by DiFraia et al.^20^ and at the MASK-air study^32^. The need for allergy apps is perceived to be higher, if a greater personal benefit can be derived from the app.

The results of this study showed that about half of the participants generally do not use pollen information services, this has already been found in other studies^29,33^. Many participants were not familiar with the availability of pollen information services. However, in developed countries, pollen information services are commonly available. This implies that public health services should disseminate information about pollen information services more widely. A part of the participants used pollen information services for planning activities, avoidance strategies as well as for planning medication intake; this is also described in the other studys^33^. However, participants did not use the APOLLO app for these purposes. In order to perform AR or AA self-management, patients need access to pollen information services, which is what the majority of patients aim for as well^33^.

### Strength and limitations

In this pilot study, we asked participants to document their complaints in the APOLLO app on a daily basis. It was not possible to query all complaints that may occur in allergy patients and to present them in an index. Skin complaints or changes in perception and behaviour could not all be surveyed due to high variability. AR is expressed very differently in patients^16^; according to this, the app is not relevant to all allergy patients to the same extent. The indices (SI; II) were formed from the individual complaints entered daily by the users. These indices were developed for this app and have not been validated. The main purpose of these indices is to provide a more simplified representation of users' daily complaints.

In addition, we were not able to perform a standardized allergy test on all participants in advance, thus these data could not be included in our analyses. Data on the presence of allergies were entirely self-reported.

Due to the location-based character of the ePIN pollen monitors, only a population from Bavaria was able to test the app. In the pilot study, only a small population was included, which was quite homogeneous: the participants were selected from an university clinical setting complemented by participants who could be recruited through offers by allergy information. They were well educated, tended to be younger and were generally in good health. This group uses health apps the most frequent^34^. In addition, the results on the health benefits of the APOLLO app must be considered in light of the fact that there was no control group. All people who registered for the study also used the APOLLO app. This was done in order to obtain as many test results as possible.

Furthermore, the app is only useful for patients, who also have a sufficiently strong interest in dealing with their allergy, either due to the severity of their complaints or their own motivation. Otherwise, the daily entries of complaints and medication rapidly lead to discontinuation of such an app^34^. Allergy self-management also brings risks, such as missing doctor visits, which leads to new recommendations being overseen, also resulting in a decrease in patients’ health^28^. Nevertheless, self-management plays a major role in therapy of chronic diseases^35^.

Pollen forecasts are of poor quality in many places and can still be significantly enhanced^36^. With the ePIN monitors real-time pollen data is measured. In addition, participants can select from eight pollen monitors throughout Bavaria. As the option to enter complaints is limited to the last three days only, the probability of recall bias is low. Based on the feedback of the participants, the menu navigation was adapted, the update of pollen concentrations by itself can be displayed in an additional section, and the complaint questionnaire was updated. The inclusion of the target group is one of the most important quality criteria in the design of a health app^37^. In general, the app meets several criteria which Krebs et al. (2015) identified in their review about good health apps, such as it is free of charge, complies with all privacy guidelines, and was evaluated as simple and comprehensive.

In order to be able to react appropriately to the individuality of AR, a self-learning app would be desirable in the future, which can provide an individual prediction of complaints of a participant based on already documented complaints and the measured pollen concentration of the ePIN monitors.

### Conclusion

The pilot study indicates that patients with pollen-related allergic rhinitis (AR) and allergic asthma (AA) are able to use the APOLLO app to learn more about their allergy and the current pollen count. This allows possibilities to adapt behaviour to the respective pollen concentration, especially for allergy sufferers with severe complaints. Self-management plays a major role in therapy of chronic diseases. However, apps for allergy management can only be one part of AR or AA therapy; a physician should always be involved. Even if only a small sample tested the app, the participants evaluated the APOLLO app as a helpful tool, which they would recommend to their families and friends. Following the pilot phase, the app will be freely accessible to the Bavarian population. For non-smartphone users, the functions of the app will also be provided as a website.

## Methods

### Requirements of the APOLLO app

When designing the app, we focused on (1) the content of the input screen and on (2) the features and navigation within the app.

Based on a literature review conducted at the beginning of the APOLLO study, we identified the complaints that participants should fill into the app on a daily basis. The primary focus was on the most common complaints of AR and AA patients. The questions in the app have been designed to be suitable for children aged 12 years and older.

When concepting the app, attention was given to choosing a simple and comprehensible interface. The app should be available to participants in both the Apple app store and the Android playstore. As the app queries and stores health data, a robust data protection concept had to be developed.

### Pollen data provided by the APOLLO app

The pollen data was obtained from the electronic pollen information network of Bavaria (ePIN). ePIN is a measuring network for recording pollen flight in Bavaria, Germany. It consists of eight electronic pollen monitors (BAA500, Helmut Hund GmbH) in Altoetting, Feucht, Garmisch-Partenkirchen, Hof, Marktheidenfeld, Mindelheim, Munich and Viechtach. The electronic pollen monitors provide data on pollen concentrations of allergenic plants in 3-h intervals free of charge^38^. ePIN is part of the Bavarian climate adaptation strategy (BayKLAS) and is implemented on behalf of the Bavarian State Ministry of Health and Care by the Bavarian Authority for Health and Food Safety.

### Pilot testing and evaluation: recruitment and participants

In spring 2021, we recruited study participants in several ways. Firstly, flyers advertising the study were distributed by HCPs of the (1) Institute and Clinic for Occupational, Social and Environmental Medicine (2) the Department of Otorhinolaryngology (3) Department of Dermatology and Allergy and (4) the University Children’s Hospital of the Ludwig-Maximilians-University (LMU) Munich. Secondly, flyers were sent to allergists, dermatologists, pulmonologists, and otolaryngologist throughout Bavaria with the request to pass them on to their patients. Addresses of specialists were obtained from the patient information system of the Bavarian Medical Association. Thirdly, we posted a digital version of the APOLLO flyer on the ePIN website (epin.bayern.de). In addition, attention was drawn to the study via various social media channels, journal articles and newsletters (Newsletter of the Allergy Information Service, Facebook page of the German Society for Allergology and Clinical Immunology, Article in the Bavarian Medical Journal^39^).

After their registration, participants gave informed consent to take part in the study. For underage participants, parental consent was obtained. Criteria for participating in the study were the presence of a pollen allergy, the minimum age of 12 years, living predominantly in Bavaria and having access to a smartphone (iOS or Android). Subsequently, the participants received the access data for the app; without access data, in this pilot phase it was not possible to run the app. The Ethics Committee at the Medical Faculty of Ludwig-Maximilian-University Munich, LMU approved the study including its data protection procedures (project nr.: 20-1149). In addition, we are confirming that all experiments were performed in accordance with relevant guidelines and regulations. The Bavarian State Ministry of Health and Care funded the APOLLO study.

### Course of the APOLLO study

To test the APOLLO app, we conducted the APOLLO study. As shown in Fig. 3, the study consists of two questionnaires and the APOLLO app. First of all (point in time T_1_), questionnaire 1 (Q1) was sent to participants, including the privacy policy and consent form. Based on the information from Q1 on the existing pollen allergy, the participants were assigned to the APOLLO app for a period of 60 days (point in time T_2_). Pollen data from previous 10 years of ePIN were analysed for this purpose before the study. This data was used to determine the main period of the respective pollen count. Even though the app was ideally supposed to be completed daily, it was possible to put in data for up to two days retrospectively. At the end of the 60 days, participants were sent questionnaire 2 (Q2, point in time T_3_).

For the questionnaires, we used already evaluated and standardized questionnaires^40–42^. When necessary, we developed additional questions based on the subject-specific knowledge of the APOLLO study team (consisting of physicians, epidemiologists, psychologists and health scientists). Figure 3 shows an overview of all topics that were part of the questions used in the APOLLO study.

### Statistical analysis

T-tests were used to compare groups (asthmatics/non asthmatics, male/female). Possible associations were analysed with linear regression models. P-values less than 0.05 were considered statistically significant. As this is a pilot study, no power calculation was carried out. No adjustments were made for missing data. All data analysis was done by using SAS 9.4 (SAS Institute, NC).

## Data Availability

Pollen data are available on ePIN.bayern.de; the health data analysed during the current study are not publicly available due to the possibility of de-pseudonymization, but are available from the corresponding author on reasonable request.
